# The Impact of Positive Verbal Rewards on Organizational Citizenship Behavior—The Mediating Role of Psychological Ownership and Affective Commitment

**DOI:** 10.3389/fpsyg.2022.864078

**Published:** 2022-04-28

**Authors:** Xin Zhao, Yi-chun Yang, Gexin Han, Qiao Zhang

**Affiliations:** ^1^School of Tourism Management, Hainan Tropical Ocean University, Sanya, China; ^2^Division of Business and Management, United International College of Beijing, Normal University-Hong Kong Baptist University, Zhuhai, China; ^3^School of Economics and Management, Zhongyuan University of Technology, Zhengzhou, China

**Keywords:** positive verbal rewards, OCB, psychological ownership, affective commitment, cognitive evaluation theory

## Abstract

Organizational citizenship behavior (OCB) can foster organizational competitiveness and survival especially, facing a rapidly changing environment. There are some empirical pieces of research that shed light on the effects of OCB on extrinsic rewards, since OCB, through performance appraisal, affects extrinsic rewards which will influence OCB as well. However, researchers have overlooked the reverse effect of extrinsic rewards (i.e., positive verbal rewards) on OCB. It is necessary to explore the mechanism between positive verbal rewards and OCB. This study integrated psychological ownership and affective commitment to form a structural model based on social exchange theory and cognitive evaluation theory. These results show that positive verbal rewards are positively correlated with psychological ownership, psychological ownership is positively correlated with affective commitment and OCB, and affective commitment is positively correlated with OCB. As refers to the mediating effects, psychological ownership fully mediates the relationship between positive verbal rewards and affective commitment. Furthermore, affective commitment plays a partial mediating role in the effect of psychological ownership on OCB. Accordingly, this psychological mechanism between positive verbal rewards and OCB reveals important theoretical and practical implications.

## Introduction

Building on the social exchange theory, organizational citizenship behavior (OCB) is a voluntary behavior of employees that goes beyond their job responsibilities and is not explicitly regulated by the company’s regular rewards systems (Paillé, 2013). This behavior is conducive to creating a positive social environment, promoting the innovation capacity of work teams, attracting and retaining talents, and improving the ability of organizations to adapt to environmental changes. When an enterprise is faced with a complex and changeable market environment, employees should go beyond their formal job responsibilities and make extra efforts to benefit the organization and enhance the core competitiveness of the enterprise. There have been a large number of research results focusing on the influencing factors and outcomes of OCB, but there are few pieces of research exploring the mechanism of motivation and OCB from the psychological perspective.

Organizational rewards, also called extrinsic rewards, have been seen as outcomes of OCB. Many empirical studies revealed that employees who constantly participate in OCB have more possibility to receive organizational rewards, such as promotions, salary increasement, appreciation praise, and special identification (Park and Sims, 1989; Podsakoff et al., 1993; Allen and Rush, 1998; Kiker and Motowidlo, 1999; Bergeron et al., 2013). On the other hand, organizational rewards have effects on employees’ intrinsic motivation. Hennessey and Amabile (2010) state that “Rewards can actually enhance intrinsic motivation and creativity when they confirm competence, provide useful information in a supportive way, or enable people to do something that they were already intrinsically motivated to do.” As a result, intrinsic motivation influences individuals’ behavior (i.e., OCB), which has been proved by psychological and neurological pieces of literature (as cited in Chen et al., 2019; Broeck et al., 2021). There is a bidirectional relationship between OCB and organizational rewards. OCB, which is related to performance appraisal, impacts rewards and is influenced by extrinsic rewards as well. Only a few organizational and motivation research indicate that extrinsic rewards enhance OCB (Johnson and Lake, 2019). The reverse mechanisms between OCB and extrinsic rewards need more thorough research.

The use of rewards and the subsequent outcomes (i.e., performance) contain many levels of paradox. Tangible incentives, such as pay for performance or contingent rewards, are often considered to be the most effective way to motivate and retain employees. However, research on cognitive evaluation theory (CET) suggests that tangible rewards and other external incentives, such as competition and performance evaluation can undermine creativity, cognize, and problem-solving abilities related to intrinsic motivation (Smith, 1975; McGraw, 1978; Amabile et al., 1990). Moreover, Hua et al. (2019) revealed that financial incentives damage the intrinsic motivator if the employees feel they are being controlled by the external interventions of the organizations. Because it is proved that performance-based monetary rewards which are perceived as controlling feedback will decrease intrinsic motivation, whereas positive verbal rewards associated with employees’ competency and accomplishment will enhance intrinsic motivation and work engagement under the CET (Pittman et al., 1980; Konstanze et al., 2014). As mentioned above, tangible rewards, such as monetary rewards, cannot always provide positive results to organizations. Since positive verbal rewards are cost-saving and effective incentives compared to economic rewards in organizational settings, the effect of intangible rewards, especially positive verbal rewards, has been worthy to be evaluated to supplement the research on motivation.

Even though theory empirical research examines the effect of extrinsic rewards on OCB, we know little about how positive verbal rewards affect employees’ OCB. Extant literature only focuses on the effects of positive verbal rewards on motivation, physiological responses, perceptions, and behavior (Konstanze et al., 2014; Hewett and Conway, 2016; Andersen et al., 2018; Brooks et al., 2019). In addition, previous research demonstrated that there is no direct relationship between extrinsic rewards and OCB (Koch and Dixon, 2007). On the one hand, rewards affect employees’ behavior by facilitating their psychological needs, psychological ownership brings us a new point of view to explain the mechanism between positive verbal rewards and OCB. Psychological ownership is a link between rewards and behavior (Qiu and Bei, 2015) and can predict extra-role behavior (namely, OCB) (Vandewalle et al., 1995; Alhadar and Hidayanti, 2021). On the other hand, organizational commitment, especially affective commitment, plays a mediation role between psychological ownership and OCB (Vandewalle et al., 1995). When employees have a strong attachment to the organization (high degree of affective commitment), they will likely to perform OCB (Organ and Ryan, 1995; LePine et al., 2002; Meyer et al., 2002; Becker and Kernan, 2003; Cropanzano et al., 2003; Zhao et al., 2019).

Since OCB is not included in the company’s explicit reward system, can we start with intangible incentives (positive verbal rewards) to make up for and improve the company’s incentive system to encourage OCB? Can positive verbal rewards motivate employees to exhibit more persistent OCBs? Therefore, the influence of positive verbal rewards on OCB and its psychological mechanism is worthy of further discussion. To our knowledge, researchers overlooked the relationship between positive verbal rewards and OCB, and the possible psychological interpretation of the relationship. To address these gaps, it is necessary to integrate positive verbal rewards, psychological mechanisms (psychological ownership and affective commitment), and OCB to form a sound construct model to help us understand the complex mechanism between these effects. Based on CET and social exchange theory, this study aims to explore the relationship between positive verbal rewards on employee’s OCB and to bring in psychological ownership and affective commitment to verify how positive verbal rewards influence an employee’s OCB through psychological ownership and affective commitment. The mediating effect of affective commitment on the influence of psychological ownership on OCB is also discussed.

This research contributes to organizational and motivation research from a different perspective. First, this study extends our understanding of the possible antecedent of OCB and the possible outcome of positive verbal rewards by examining the effect of positive verbal rewards on OCB. Building on social exchange theory and CET, we proposed an integrated model to leverage an understanding of the mechanism between positive verbal rewards and OCB. This study also contributes to psychological literature by, second, explicitly demonstrating the role of psychological ownership and affective commitment associated with positive verbal rewards and OCB. Finally, this research contributes to academic studies on human resource management. To cultivate employees’ OCB, traditional paradigms only focus on factors from an organizational and personal level, or material extrinsic rewards, this study provides that a reward system with intangible rewards will cultivate employees’ OCB and that managers should pay more attention to employees’ psychological needs.

## Literature Review and Research Background

### Cognitive Evaluation Theory

Cognitive evaluation theory (CET), developed by Deci and Ryan (1985), states that feelings of self-determination and competence are two key psychological needs underlying intrinsic motivation. The effects of rewards depending on how the recipients interpret the rewards. Accordingly, if an event, such as a reward satisfies these psychological needs, it can enhance intrinsic motivation by increasing the perceived self-determination and perceived competence. Whereas failure to meet these needs will reduce intrinsic motivation. CET further asserts that extrinsic rewards influence intrinsic motivation in two different ways: controlling or informational aspects (Ryan et al., 1983). For example, monetary rewards are expected to decrease intrinsic motivation because they reduce the feeling of self-determination and prompt a change in the perceived locus of causality. In contrast, positive verbal rewards, perceived to be informational and supportive, tend to facilitate intrinsic motivation by enhancing the feeling of competence when doing a job (Deci et al., 2001).

Cognitive evaluation theory (CET) argues that, just like intangible motivation, the interpersonal context created by positive feedback influences how people perceive it, which in turn affects the effectiveness of motivations. Two studies confirmed that controlling positive feedback elicits less intrinsic motivation than informative positive feedback. An example of controlling feedback is the feedback that contains the word “should,” such as “Great, you should keep performing well,” which means putting pressure on the person who receives the feedback to keep doing their job well (Ryan, 1982). This is, in contrast, to simply giving participants feedback on their scores and indicating that they performed well above the average. In another study, such as “I can’t recommend you for promotion yet, but you are doing a good job and if you insist, I will have a chance to recommend you to the boss.” Such verbal statements are controlled feedback. “Compared to most of my subjects, you did very well.” It’s a statement of informational feedback. Another implication of controlling feedback is that the manager needs the participant to do well, and the participant feels pressure. In both studies, informational feedback elicited more intrinsic motivation than controlling feedback. Accomplishment, namely, positive verbal rewards, contains informational components. It is said that positive verbal rewards facilitate intrinsic motivation.

### Social Exchange Theory

Social exchange theory can be used as a basic model to understand organizational behavior (Roch et al., 2019). Its core principle is the principle of reciprocity. Employees’ behavior is, based on the social costs and rewards evaluation, a social form of exchange (tangible or intangible). Only if the perceived value of the exchange is positive, the relationship can be retained. The exchange relationship between organizations and employees is often illustrated by social exchange theory (Chernyak-Hai and Rabenu, 2018). Existing studies have demonstrated the explanatory role of social exchange theory and well explained various interactions between employees’ work attitudes and behavioral results (Conway and Briner, 2005; Chia-An Tsai and Kang, 2019). The reciprocity principle is based on the assumption that members of an organization tend to reward their behavior with work-related behavior and retaliate for negative treatment they receive at work (Gouldner, 1960). In addition, social exchange theory also shows the repeatability and coherence of individuals, that is, constantly seeking fair and balanced exchange (Cropanzano and Mitchell, 2005). This principle of reciprocity is therefore extremely valuable in attracting, retaining, and motivating employees.

## Research Hypotheses

### Positive Verbal Rewards and Psychological Ownership

The term positive verbal rewards is not commonly used in the current literature on intrinsic motivation. The term “positive verbal rewards,” often referred to as “positive feedback,” was used to make it easier to incorporate positive feedback research into the general category of motivation and thus compare their effects with tangible rewards. In this regard, one of Deci’s initial studies found that positive feedback can enhance intrinsic motivation (Deci, 1971). According to CET, the informational side of positive verbal rewards is generally considered to be prominent, so positive verbal rewards are usually predicted to enhance intrinsic motivation.

The core of psychological ownership is a sense of possessiveness and the feeling of psychological involvement of the target (Pierce and Peck, 2018). For employees, psychological ownership makes them have the belief that “they are the owner of the enterprise,” and this psychological perception makes the employment relationship more solid. Pierce et al. (2003) argue that ownership is: (1) innate; (2) psychological ownership occurs on tangible or intangible objects; and (3) psychological ownership produces a psychological, attitudinal, and behavioral response to actual ownership. Employees’ feelings of possession and psychological connection to an organization can lead to a strong belief and acceptance of organizational goals and values (Meyer et al., 1993). Simard et al. (2005) revealed that some rewards, such as recognition and ability development, can stimulate employees’ psychological connection to the organization. Specifically, the recognition system enables the organization to express its appreciation for high-quality work and achievements to employees clearly. Therefore, when employees feel that their abilities, efforts, and performance contributions are recognized by the organization, they will show greater psychological ownership (Davies, 2001).

According to CET, feelings of competence and autonomy are both important for intrinsic motivation. Research has shown that positive verbal feedback satisfying the needs of competence and autonomy promotes intrinsic motivation and promotes a sense of competence when people feel responsible for their successful performance (Deci, 1971; Fisher, 1978; Ryan, 1982; Burgers et al., 2015). Social exchange theory further shows that when managers are willing to provide care for employees and care about their needs, positive exchange relationships will occur (Allen et al., 2003). Therefore, this study makes a hypothesis that positive verbal rewards can promote employees’ psychological ownership because employees have feelings of the company’s support, trust, and commitment to their employees (Eisenberger et al., 1986; Guzzo and Noonan, 1994). Accordingly, the following hypothesis is proposed:

H1: positive verbal rewards has a significant positive impact on employees’ psychological ownership.

#### Psychological Ownership and Affective Commitment

Academic research has confirmed that people will also have feelings of ownership for non-material subjects (artworks, concepts, thoughts, relationships, people, etc.) (Pierce et al., 2001). Furby (1978); Dittmar (1992) illustrated that employees’ psychological ownership of the target would make them think that the target is an extension of themselves and produce a self-concept. Furby (1978) stated that the feeling of power would make people feel responsible for the object. Avey et al. (2008) believe that psychological ownership represents a multidimensional structure, originating from self-efficacy, sense of responsibility, and sense of belonging and identity. People will protect and defend their own ownership because of psychological ownership. Therefore, psychological ownership will affect people’s attitudes, motivation, and behavior (Pierce et al., 1991; Van Dyne and Pierce, 2004). Employees often have a sense of ownership of their own ideas and thoughts, and therefore naturally have a sense of ownership of their projects or tasks.

Affective commitment is one of the three components of organizational commitment established by Meyer and Allen (1991). It has received more attention than continuance and normative commitment because it can predict the psychological behavior of employees (Parris and Peachey, 2012; Jackson et al., 2013; Ling et al., 2017). It is defined as follows: “an affective or emotional attachment to the organization such that the strongly committed individual identifies with, is involved in, and enjoys membership in, the organization” (Allen and Meyer, 1990). It is a psychological feeling of belonging and attachment when employees hold the same values and goals as the organization. As a result, affective commitment increases employees’ loyalty and involvement in the organization. Previous research on affective commitment shows that organizational support, organizational justice, autonomy, work experiences, and personal characteristics are antecedents of affective commitment (Allen and Meyer, 1990; Mathieu and Zajac, 1990). In addition, affective commitment is related to performance, turnover intentions, and absenteeism (Mercurio, 2015).

Psychological ownership refers to a psychological feeling of possession, it is supposed to have more correlation with affective commitment than continuance and normative commitment (Mayhew et al., 2007). Existing studies on organizational behavior have confirmed that psychological ownership has an impact on employees’ affective commitment to their organizations and employees’ extra-role behaviors (Vandewalle et al., 1995; Pierce et al., 2001; Avey et al., 2009; Nancy et al., 2009; Bernhard and O’Driscoll, 2011). Once employees have a strong sense of psychological ownership, employees can better contribute to the organization and have a deeper emotional attachment to the organization. Pierce et al. (2001) believes that the intensity of employees’ psychological ownership is, in a sense, mainly determined by their dependence on the organization. Therefore, only when employees think “this is my organization,” will they become attached to the organization and establish a strong identification and commitment to the organization emotionally. In other words, the enhancement of the perceived strength of psychological ownership will further promote the perceived strength of affective commitment of employees.

Psychological ownership is a kind of positive psychology attachment, which can effectively show the specific situation of employees’ commitment to the organization. Many scholars believe that psychological ownership has positive associations with affective commitment (Kwak et al., 2012; Zhang et al., 2021). Therefore, if employees have a higher-level sense of ownership (psychological ownership) with the organization, they will have a higher level of affective commitment. Through their sense of ownership, employees see the organization as their “home,” a place that provides psychological comfort and security. To sum up, this study proposes the following hypothesis:

H2: Psychological ownership of employees has a positive impact on affective commitment.

#### Organizational Citizenship Behavior and Psychological Ownership

Organizational citizenship behavior (OCB) can be defined as the extra-role behavior of individuals in the workplace, which is not explicitly recognized by the formal reward system (Organ et al., 2006; Jain et al., 2011). In other words, OCB is the way people choose to do beneficial behaviors for others (Peloza and Hassay, 2006). These extra-role behaviors may contribute to the construction of social and psychological environments, thus contributing to the execution of tasks in organizations (Organ, 1997). Based on the reciprocity derived from the social exchange theory, employees may take voluntary behaviors to repay the organization (Organ et al., 2006). That is to say, employees who act as citizens of the organization go above and beyond the role required by the organization, and these behaviors ultimately benefit the organization. Because of its importance in promoting organizational efficiency and success, OCB has been extensively studied on many issues.

Psychological ownership can stimulate the extra-role behavior of employees, and also can stimulate the altruistic behavior expected by the external role. The research of Vandewalle et al. (1995) shows that psychological ownership is highly correlated with OCB, and psychological ownership can be used to predict the OCB of employees. The research of Furby (1991) also found that there is an inseparable relationship between psychological ownership and OCB. He argues that psychological ownership determines job satisfaction. In addition, Van Dyne and Pierce (2004) and O’Driscoll et al. (2006) also found a positive correlation between psychological ownership and OCB. Consistent with previous research, this study proposes the following hypothesis:

H3: Psychological ownership has a significant positive impact on employees’ OCB.

#### Affective Commitment and Organizational Citizenship Behavior

Affective commitment, related to positive experiences, is an important predictor of employees’ behavior (Dahleez et al., 2020). As a result, many studies have shown that affective commitment is a special psychological dynamic process of employees toward the organization, which can predict OCB very well (Eisenberger et al., 1990; Shore and Wayne, 1993; Morrison, 1994; Organ and Ryan, 1995; LePine et al., 2002; Meyer et al., 2002). From the perspective of social exchange theory, an employee with highly affective commitment is likely to reciprocate to the organization by performing OCB (Cropanzano et al., 2003). Especially, Chen and Francesco (2003) found that affective commitment positively influenced employees’ OCB. Feather and Rauter (2004) further found a strong positive correlation between affective commitment and OCB of teachers by studying the perceived strength of teachers’ affective commitment. Extensive research has confirmed that affective commitment positively affects employees’ OCB. Therefore, the following hypothesis is proposed in this study:

H4: Affective commitment has a significant positive impact on OCB.

#### The Mediation Role of Affective Commitment

The studies of many scholars have proved that affective commitment plays a significant mediating role between psychological ownership and OCB (Vandewalle et al., 2005). Affective commitment plays a partially mediating role in the impact of performance evaluation and OCB (Li et al., 2010), perceived CSR and OCB of employees (Liu and Zhou, 2015), human resource management intensity, and service-oriented OCB (Zhu et al., 2020). It is confirmed that affective commitment is a mediating variable between psychological ownership and OCB. Therefore, this study continues to take affective commitment in organizational commitment as a mediator variable and take OCB as a consequence variable to test the relationship among all constructs in the context of Chinese culture. This study will analyze and test the following assumption:

H5: Affective commitment plays a partially mediating role in the influence of psychological ownership and OCB.

#### The Mediation Role of Psychological Ownership

Psychological ownership, reflecting the target of ownership, is an important predictor of organizational effects, such as organizational commitment or OCB (O’Driscoll et al., 2006; Avey et al., 2009; Pierce and Peck, 2018). Empirical studies show that PO has a positive influence on the way individuals behave in organizational settings. It has predictive power on OCB (Organ, 1988) over the effects of organizational commitment (Liu et al., 2011). In addition, psychological ownership as a unidimensional construct helps us explain the motivation of employees. Work practices, such as positive verbal rewards, will lead employees to hold a sense of control and experience psychological ownership. Furthermore, the sense of ownership will motivate employees to develop affective commitment and participate in OCB (Liu et al., 2011). As a result, psychological ownership mediates the relationship between positive verbal rewards and affective commitment.

H6: Psychological ownership mediates the relationship between positive verbal rewards and affective commitment.

## Methodology

### Samples and Procedure

An online questionnaire survey was designed to test the proposed construct model. For validity and reliability concerns, the measurement of all variables was developed from existing literature and revised based on the purpose of the research. The scales in this research are originated from English countries, hence the back-translation technique was applied. The measurement was translated to Mandarin and then back-translated to English. Three professors working on organizational behavior and psychology, and two supervisors who worked in the service industry were invited to validate the scale. In addition, gender, age, educational background, income, and seniority were taken as control variables because these demographic factors may impact OCB (Allen and Jang, 2018).

We searched the hotels in Macau from Tripadvisor (an online OTA platform), then 150 hotels were displayed as the results. After inserting the names of 150 hotels into Excel, the Random Number Generators of SPSS were applied to select a 16% random sample of cases. A total of 25(16%) hotels were finally selected as target hotels where the data collection processes were implemented. Hotel employees in Macau were selected as research objects because hotel chains have a well-designed organizational structure and qualified employees. The questionnaire was sent out through working groups under the organizations’ office automation systems or any other organizational internal social network platforms. Instructions on the research and monetary reward were included to improve the response rate. A pretest to 30 respondents was conducted to make sure the content validity. The final questionnaire was modified to ensure validity and reliability.

### Measurements

The construct was measured on a 7-point Likert-type scale, ranging from 1 (strongly disagree) to 7 (strongly agree). The questionnaire aims to evaluate the positive verbal rewards, psychological ownership, affective commitment, OCB, and background information of respondents.

Positive verbal rewards. This study defined positive verbal rewards as all positive verbal or written praise, accomplishment, recognition, affirmation, etc. The positive verbal rewards items were based on a 3-item scale developed by Andersen et al. (2018). Sample items included “My organization gives employees positive feedback when they perform well,” “Actively shows appreciation of employees who do their jobs better than expected,” and “Personally compliments employees when they do outstanding work.”

Psychological ownership. Pierce et al. (2001) believe that psychological ownership is a psychological state in which employees in an organization feel the tasks of the organization as if they were their own tasks. The five measure items of psychological ownership in this study were adapted from Pierce et al. (2001), such as “I feel a very high degree of personal ownership for this organization.”

Affective commitment. Affective commitment is defined as the emotional tendency of employees to identify with organizational values and goals, to be loyal to the organization, to have a strong emotional dependence on the organization, and to work hard and devote themselves to the development of the organization. For the measurement of affective commitment, this study adopted a 5-item scale developed by Meyer and Allen (2007). Sample items included “I am proud to tell others that I am a part of this organization” and “I feel personally attached to my work organization.”

As for the measurement of OCB, Jiing-Lih Farh et al.’s (2004) scale is the most widely used because it was developed in the Chinese context. This scale contains eleven dimensions, which are divided into self-level, interpersonal level, organizational level, and social level. On this basis, the final research scale is made up of three aspects: self-level, interpersonal level, and organizational level, including five dimensions of taking initiative, helping coworkers, interpersonal harmony, promoting company image, and group activity participation, including 20 questions. These factors form the second-order construct. Taking initiative refers to an individual’s willingness to take on extra-role responsibilities (e.g., “volunteer for overtime work”). Helping coworkers means helping coworkers with work-related or non-work matters (e.g., “help coworkers in non-working matters”). Interpersonal harmony refers to employee actions aimed at facilitating and preserving harmonious relations in the workplace (e.g., “maintain harmonious relationships and diffuse conflict”). Promoting company image means loyalty to the organization and sharing the same value and objectives with the organization (e.g., sharing useful work-related information). Group activity participation refers to participating in an organization’s activities (e.g., “participate in company-organized group activities”).

## Results

### Descriptive Statistics

A total of 377 employees replied to the survey. After removing invalid questionnaires (incomplete information, those who didn’t match the attention check, or select the same option, and the time of completion were below the average completion time), 312 valid questionnaires were obtained. Table 1 shows that the majority of respondents are women and about two-fifth of these are younger than 35(40.06%). Over 50% of the respondents have a bachelor’s or master’s degree and have at least 3 years of working experience. In sum, the participants were young, better educated, and experienced employees.

**TABLE 1:** Demographic characteristics of respondents (*N* = 312).

Item	category	Frequency	Percentage
Gender	Male	132	42.31%
	Female	180	57.69%
Age (years)	<25	12	3.85%
	25–34	113	36.21%
	35–44	127	40.71%
	45–54	56	17.95%
	>55	4	1.28%
Educational level	Middle school and below	4	1.28%
	High school	30	9.62%
	3-year college	108	34.62%
	Bachelor’s degree	162	51.92%
	Master’s degree and above	8	2.56%
Seniority	<1 year	24	7.69%
	1–3 years	52	16.67%
	3–5 years	121	38.78%
	5–10 years	103	33.01%
	>10 years	12	3.85%

### Measurement Model Analysis

First, the model was verified *via* partial least squares using SmartPLS 3.0, which was mainly used to test the loadings, the composite reliability (CR) of each facet, average variance abstraction (AVE), and model adaptation. This study refers to the criteria given by Hair et al. (2019). As shown in Table 2, the factor loadings of all the items (including second-order measurements) in this study are between 0.716 and 0.887, and if it is greater than 0.7, it means that all the latent variables have explanatory ability to the observed variables. The AVEs were above 0.5, and the CRs were greater than 0.7. The results indicated that this construct has good convergent validity. The discriminant validity, according to Fornell and Larcker (1981) criterion, was demonstrated because the square roots of AVE for all constructs exceeded inter-construct correlations of the factors (Table 3). Moreover, the values of variance inflation factor (VIF) (Table 4), through the VIF test, are lower than 3.3 (Kock, 2015; Hair et al., 2019), suggesting that this study is free of multicollinearity. At last, due to the single source of the data, the issue of common method bias was evaluated by Harman’s single-factor test. The results show that common method bias was not to be a concern in this study, because the single factor accounted for 40% of the variance (lower than 50%).

**TABLE 2:** Result of the measurement model.

Factors	Items	Loading	*T*-value	Cronbach α	AVE	CR
Positive verbal rewards				0.939	0.587	0.850
	PR1	0.778	13.033			
	PR2	0.788	11.808			
	PR3	0.716	12.907			
Psychological ownership				0.946	0.756	0.939
	PO1	0.862	19.404			
	PO2	0.878	20.068			
	PO3	0.887	20.465			
	PO4	0.859	19.285			
Affective commitment				0.942	0.656	0.905
	AC1	0.817	13.852			
	AC2	0.833	14.136			
	AC3	0.830	14.082			
	AC4	0.819	13.890			
Taking initiative (second-order)				0.913	0.672	0.891
	P1	0.855	17.269			
	P2	0.851	17.177			
	P3	0.719	13.512			
Helping coworkers (second-order)				0.921	0.671	0.891
	HC1	0.844	16.519			
	HC2	0.820	15.863			
	HC3	0.779	14.789			
Interpersonal harmony (second-order of OCB)				0.910	0.669	0.890
	IH1	0.828	15.554			
	IH2	0.845	15.945			
	IH3	0.785	14.512			
Promoting company image (second-order of OCB)				0.909	0.648	0.880
	OI1	0.840	15.484			
	OI2	0.842	15.530			
	OI3	0.725	12.908			
Participating in organizations (second-order of OCB)				0.927	0.735	0.917
	PI1	0.872	17.874			
	PI2	0.869	17.798			
	PI3	0.857	17.430			

**TABLE 3:** Analysis of discriminant validity.

Constructs	Fornell-Larcker criterion			
	Positive verbal rewards (VR)	Psychological ownership (PO)	Affective commitment (AC)	OCB
VR	0.77			
PO	0.35	0.87		
AC	0.47	0.41	0.81	
OCB	0.51	0.57	0.55	0.89

**TABLE 4:** Collinearity scores based on the variance inflation factor test.

	OCB	Positive verbal rewards	Psychological ownership	Affective commitment
**OCB**				
Positive verbal rewards	1.217			
Psychological ownership	2.033			
Affective commitment	1.966			
				

**FIGURE 1:**
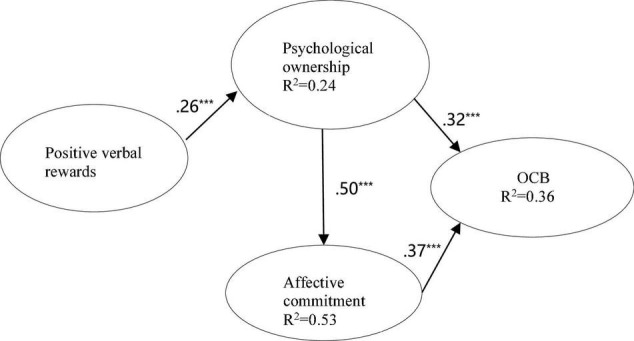
Structural model testing results. ***p < 0.001.

As shown in Figure 1, the structural model was tested using SmartPLS 3.0. Positive verbal rewards significantly impact psychological ownership (β = 0.26, *p* < 0.001) and explained 24% of the variance in the construct, thus supporting H1. Psychological ownership (β = 0.50, *p* < 0.001) positively related to affective commitment and explained 53% of the variance in affective commitment, supporting H2. Psychological ownership (β = 0.32, *p* < 0.001) and affective commitment (β = 0.37, *p* < 0.001) explained 36% of the variance in OCB, providing support for H3 and H4.

### Hypotheses Testing

The proposed hypotheses were assessed by the path coefficients. It can be seen from Table 5 that all the hypotheses are significant when *p* < 0.001. In other words, positive verbal rewards positively affect employees’ psychological ownership. Psychological ownership has a positive impact on OCB, while affective commitment has a positive impact on OCB. OCB has a significant impact on taking initiative, helping coworkers, interpersonal harmony, organizational identification, and group activity participation. That is, all paths of the proposed model are reasonable and all research hypotheses are supported.

**TABLE 5:** Results of measurement model.

From	To	Path coefficient	*T*-value	*P*-value	Results
Positive verbal rewards	Psychological ownership	0.318	4.651	0.000	Supported
Psychological ownership	Affective commitment	0.383	7.656	0.001	Supported
Psychological ownership	OCB	0.206	4.251	0.000	Supported
Affective commitment	OCB	0.303	4.541	0.000	Supported

### Mediating Effect

In this study, the bootstrapping procedure with confidence intervals bias-corrected 95% was used to test the mediating effect of psychological ownership on the relationship between positive verbal rewards and affective commitment and the mediating effect of affective commitment on the relationship between psychological ownership and OCB, based on 5,000 bootstrap samples (Hair et al., 2019). As indicated in Table 6, at *p* < 0.01, both direct and indirect effects are significant. Therefore, H5 and H6 are proved to be true. That is to say, psychological ownership fully mediates the relationship between positive verbal rewards and affective commitment. Moreover, affective commitment has a partial mediating effect on the relationship between psychological ownership and OCB. That is to say, if employees who receive positive verbal rewards will have a high sense of psychological ownership and then their affective commitment increases. Furthermore, psychological ownership-affective commitment-OCB indirect linkage exists. This direct and indirect linkage demonstrated that affective commitment partially mediates the relationship between psychological ownership and OCB.

**TABLE 6:** Direct and indirect effects of the model.

Structural paths	Direct effects	Indirect effects	Total effects
positive verbal rewards(PVR) –> psychological ownership(PO)	0.261**		
PO –> affective commitment(AC)	0.498**		
AC –> OCB	0.366**		
PO –> OCB	0.322**		
PO –> AC –> OCB		0.182**	0.504**
PVR –> PO –> AC		0.130**	

**p < 0.01.

## Discussion and Implication

Grounded on the CET and social exchange theory, this study aims to take positive verbal rewards as new antecedents of OCB and examine how positive verbal rewards influence psychological ownership and affective commitment, and OCB. This study went beyond the traditional research attention on the influence of the working environment on OCB. As expected, all the hypotheses were supported and the results are consistent with previous pieces of literature. First, the research findings indicate that the relationship between positive verbal rewards and OCB is a psychological process where positive verbal rewards positively affect employees’ OCB through psychological ownership and affective commitment. This is in line with the findings of Ilgen et al. (1979), Ellingsen and Johannesson (2008) that positive verbal rewards (i.e., praise or public recognition) have been found to be a vital motivator of work-related behaviors. Thus, positive verbal rewards are important in predicting employees’ behaviors. Second, consistent with previous literature, the results indicate that psychological ownership has a positive effect on affective commitment which can further influence employees’ OCB (Zhang et al., 2021). The triangle relationships between psychological ownership, affective commitment, and OCB are proved again. In addition, the results also confirm that psychological ownership mediates the relationship between positive verbal rewards and affective commitment and affective commitment partially mediates the relationship between psychological ownership and OCB.

In terms of the influencing effects of positive verbal rewards, praise or organizational recognition satisfies employees’ needs for competence and autonomy and enhances intrinsic motivation. That is to say, intangible daily rewards support employees’ expectation that they will gain more sense of achievement and will perform more engagement behaviors. The theoretical explanation for these mechanisms is that positive verbal rewards contain the informational aspects of competency or accomplishment which contribute to the satisfaction of psychological needs, resulting in more intrinsic motivation. Behaviors can be predicted by motivation. Thus, employees who receive more positive verbal rewards during the work process will have more feelings of ownership and emotional attachment to the organization and then participate in more OCB.

### Theoretical Contributions

This study contributes to the academic literature in several ways. First, the study investigates the reverse mechanism between OCB and positive verbal rewards and proposes an integrated construct model to clarify the complex effects. Previous research has examined different sorts of antecedents of OCB and takes positive verbal rewards as the outcomes of OCB (Park and Sims, 1989; Podsakoff et al., 1993; Allen and Rush, 1998; Kiker and Motowidlo, 1999; Bergeron et al., 2013). However, there is a bidirectional relationship between OCB and organizational rewards and positive verbal rewards can predict OCB in reverse. The scholars in the organizational behavior and psychology field have paid little attention to the role that positive verbal rewards play on psychological states and extra-role behavior. This study tends to provide a more holistic explanation of how positive verbal rewards influence employees’ psychological needs and OCB performance. The suggestion, therefore, is that positive verbal rewards are important influencers in fostering an understanding of the effects of rewards (Ross, 1975).

Second, this study makes a contribution to extending the CET. Grounded on CET, psychological (psychological ownership) and behavioral (OCB) factors were introduced to develop the integrated model and to test the impact of the positive verbal rewards. Since positive verbal rewards on performance can improve an individual’s self-esteem and self-determination to enhance intrinsic motivation (Brooks et al., 2019), the key question lies in how to use rewards to trigger and enhance intrinsic motivation. This supports the suggestion that rewards will not exert an undermining effect once rewards are delivered in an informational and supportive way rather than a controlling way (Deci et al., 2001). This research contributes to motivation literature as well.

Third, this study, conducted in China, contributes to the organizational and motivation research in the Chinese context. Most of the scales were developed in western developing countries, there might be slight differences in different cultures. The back-translate technique and scale of OCB designed in Chinese culture were used to ensure reliability and validity. This study on how extrinsic rewards can lead to improving employees’ OCB in the Chinese context supplements the existing studies in developing countries.

### Practical Implications

First, the study proves that positive verbal rewards have a positive effect on employees’ psychological ownership and affective commitment, which can further influence employees’ OCB. Therefore, enterprises should improve the degree of perceived psychological ownership through diversified positive verbal rewards measures. This study proposes to include the beneficial behavior of OCB into the performance assessment process and to build a career development system for employees based on their good conduct. The training focus on employees’ psychological states and attitudes can make up for the insufficiency of traditional training only focusing on professional knowledge, ability, and skills of a specific position. Particularly, organizations have to design training programs according to the demand analysis and pay more attention to psychological training for new employees, so that employees always maintain a positive working attitude, and companies achieve long-term development. Through the efficient communication system, employees can not only express their opinions freely, express their suggestions and dissatisfaction in time but also get timely positive verbal rewards from their superiors, solve problems in work or life, and achieve a state of wholehearted work. On the other hand, a good working environment can enhance employees’ perception of psychological ownership, make them psychologically feel corporations’ respect and trust, and has a sense that “I belong to the organization.” As a result, employees are supposed to exert more OCB.

Second, the empirical study proves that there is a positive correlation between psychological ownership and affective commitment. In addition, this study also confirms that affective commitment can also have a positive impact on employees’ OCB. Therefore, this study proposes that a “people-oriented” corporate environment which is the basis for developing psychological ownership and enhancing affective commitment should be constructed, because it can not only enhance organizations’ unique advantages in external competition but also form a kind of cohesion within the organizations. This study argues that it should include appropriate emotional management and democratic management. Emotional management should always focus on psychological perception and the ideas of employees in organizations, the employees within the organization can be blended into the enterprise’s vision and mission because the essence of organizations is also with the development of the integration of the employees in an organization. The core idea of this integration is that the organizational environment can stimulate employees’ work enthusiasm. Democratic management requires managers and supervisors to be neutral without the impact of bias interest, emotion, and listen to the employees for their advice and opinions. Thus promoting organizations build a democratic atmosphere, makes the enterprise staff speak freely so that employees perceive working in the enterprise to have feelings of home, free, and comfortable. It can also achieve the purpose of helping and supporting each other among employees, to enable employees to exercise OCB independently.

Finally, the study confirms that psychological ownership and affective commitment play a mediating role in the integrated model. Therefore, enterprises should pay attention to the cultivation and care of employees’ affective commitment, which can not only increase psychological ownership but also effectively increase the employees’ perceived affective commitment, promote employees’ emotional connection to the organization, and realize efficient OCB. This study proposes a managerial intervention, such as fault-tolerant, to encourage innovation and improve the guarantee mechanism based on OCB to ensure the effective implementation of OCB. A perfect enterprise management system should not only contain specific implementation rules but also perfect safeguard and supervision measures. Fault-tolerant mechanism and supervision mechanism can jointly promote the effective implementation of organizational citizenship behavior of employees from the aspects of psychological ownership and affective commitment.

### Limitations and Future Research

The business environment faced by organizations is changing rapidly and uncertainly, companies are expecting their employees to perform more OCB in a cost-saving way (Zhao et al., 2014; Zhang et al., 2020). Extrinsic rewards, such as positive verbal rewards, are important for predicting OCB. Besides, psychological ownership mediates the relationship between positive verbal rewards and affective commitment, and affective commitment plays a mediating role in the relationship between positive verbal rewards and OCB. This study has some limitations which suggest an avenue for future research. First, data were collected from one particular industry in a location, which influence its generalization. Future studies may examine the link in other industries and countries to expand cross-culture results. Second, this study only examines positive verbal rewards as antecedents of OCB. Future studies may further explore how different rewards (i.e., monetary rewards) affect employees’ psychological states and OCB engagement (Yang et al., 2020). Third, this study uses a single measurement data which will weaken the causality of the factors. Thus, future studies can test the construct by using the longitudinal approach to collect data.

## Data Availability Statement

The raw data supporting the conclusions of this article will be made available by the authors, without undue reservation.

## Ethics Statement

Ethical review and approval were not required for the study on human participants in accordance with the local legislation and institutional requirements. The patients/participants provided their written informed consent to participate in this study.

## Author Contributions

XZ was responsible for the analysis and interpretation of the data, drafted the manuscript, and improved the English writing. Y-CY and QZ were responsible for the conception and design of the study and revised the manuscript. GH was responsible for the design of the survey and collection of the data. All authors contributed to the article and approved the submitted version.

## Conflict of Interest

The authors declare that the research was conducted in the absence of any commercial or financial relationships that could be construed as a potential conflict of interest.

## Publisher’s Note

All claims expressed in this article are solely those of the authors and do not necessarily represent those of their affiliated organizations, or those of the publisher, the editors and the reviewers. Any product that may be evaluated in this article, or claim that may be made by its manufacturer, is not guaranteed or endorsed by the publisher.
